# Comparison of cross-sectional area and fat infiltration of suboccipital muscles between normal dogs and dogs with atlantoaxial instability

**DOI:** 10.1186/s12917-021-03132-0

**Published:** 2022-01-18

**Authors:** Namsoon Lee, Munsu Yun, Junghee Yoon

**Affiliations:** 1 Time Animal Medical Center, 57, Dunsan-ro, Seo-gu, 35233 Daejeon, South Korea; 2 College of Veterinary Medicine, Research Institute for Veterinary Science, Seoul National University, Gwanak-ro, Gwanak-gu, 08826 Seoul, South Korea

**Keywords:** AAI, MRI, Suboccipital muscles, Toy dogs

## Abstract

**Background:**

Atlantoaxial instability (AAI) is primarily a congenital neurological disorder affecting young toy-breed dogs. So far, most studies have focused on bones and ligaments related to AAI, and there are no studies on the suboccipital muscles (SOMs) that occupy a large area from the occipital bone to C2 in dogs. This study evaluated the cross-sectional area (CSA) and fat infiltration of the SOMs using magnetic resonance imaging (MRI), specifically, T1-weighted images, in normal dogs (≤ 5 kg) and AAI dogs. The relationship between the severity of the neurological symptoms of AAI (group A and group B) and the values from MRI was also assessed.

**Results:**

AAI dogs had significantly smaller CSA (*P* = 0.029) and greater fat infiltration (*P* = 0.044) of the SOMs compared to normal dogs. AAI dogs with mild neurological symptoms for a long period (group A) had greater fat infiltration than AAI dogs with severe neurological symptoms (group B) (*P* = 0.035).

**Conclusions:**

The muscle changes are most likely due to spinal cord compression resulting from instability; however, the possibility that chronic changes of the muscle may play an additional role in maintaining stability in this region cannot be excluded. This study provides fundamental quantitative information of the SOMs in normal and AAI dogs.

## Background

Atlantoaxial instability (AAI) is primarily a congenital neurological disorder affecting young toy-breed dogs, including Maltese, Chihuahua, Toy Poodle, Pomeranian, and Yorkshire Terrier [1]. Several reports describe aetiologies for AAI, including congenital or developmental causes associated with aplasia of the dens, non-union of the ossification centres, and absence of ligamentous support in toy-breed dogs, as well as trauma associated with a fracture of the dens and/or disruption of the ligamentous support in any breed of dog of any age [2, 3].

Since the occipital region of the skull and the first two cervical vertebrae (C1 and C2) develop together embryologically, many dogs are subject to multiple developmental disorders such as caudal occipital malformation syndrome and hypoplasia, atlanto-occipital overlapping, and AAI [4]. Due to its rotational movements, and to the lesser extent of lateral bending with the absence of an intervertebral disc, the atlantoaxial joint (AAJ) requires additional stabilization by suboccipital muscles (SOMs), cervical fasciae and ligamentous structures [5, 6]. Therefore, recently, more specific studies about concurrent diseases with AAI and biomechanical evaluation of the stabilizing function in this region have been conducted [6–9].

Human studies have used magnetic resonance imaging (MRI) to investigate the cross-sectional area (CSA) and fat infiltration of the SOMs to design effective management strategies for upper neck problems [10–12]. Similar studies have been conducted on the thoracolumbar (TL) epaxial muscles in veterinary medicine [13, 14], however, no studies have evaluated the SOMs occupying a large area of this region from the occipital bone to C2 in dogs using MRI.

We hypothesized that there would be a difference in the CSA and fat infiltration of the SOMs between normal dogs and AAI dogs because these muscles may also play a role in stabilizing in AAJ. Among AAI patients, these values could vary, depending on the severity of neurological symptoms. The purpose of this study was to evaluate the CSA and fat infiltration of SOMs using MRI in normal dogs (≤ 5 kg) and AAI dogs, specifically to identify whether there are differences in these values between normal and AAI dogs, and whether these values vary depending on the severity of the AAI symptoms.

## Results

Two groups of small dogs (≤ 5 kg, 26 dogs) were included in the study; the first group consisted of 13 normal dogs and the second consisted of 13 dogs with AAI. The mean age and weight of AAI and control dogs and significant differences between groups are displayed in Table 1.

**Table 1:** Breeds, mean age and weight, and significant differences between normal and AAI dogs

Group (number of dogs)		Breed (number of dogs)	Age in years (range)		Weight in kg (range)	
Normal (13)		Maltese (9), Toy Poodle (3), Pomeranian (1)	4.92 (1.6–8.0)		3.87^a^ (2.64–5.0)	
AAI (13)	A (7)	Maltese (4), Yorkshire Terrier (2), Pomeranian (1)	5.26^b^ (0.5–12.0)	7.58 (4.6–12.0)	2.64 (1.36–5.0)	2.14 (1.36–3.4)
	B (6)	Chihuahua (2), Maltese (1), Toy Poodle (1), Pomeranian (1), Spitz (1)		2.56 (0.5–4.0)		3.22 (1.4–5.0)

^a^Significant difference in weight between normal and AAI dogs (*P* = 0.005). ^b^Significant difference in age between group A and B of AAI dogs (*P* = 0.001)

All dogs were of toy-breeds, and Maltese was the most common breed in AAI dogs. Normal dogs weighed more than AAI dogs (*P* = 0.005). The AAI dogs were also split into two groups based on severity of the neurological symptoms, where group A (mild symptoms) of AAI dogs were older than group B (severe symptoms) (*P* = 0.001). The duration of neurological symptoms before admission ranged from two days to 4.5 years. The duration of group A (mean: 1.35 years, range: two months to 4.5 years) was longer than that of group B (mean: 130 days, range: two days to six months) (*P* = 0.008). A history of trauma was reported in two dogs. Abnormalities of the dens were identified in eight dogs, and seven of these showed congenital anomalies.

**Fig. 1:**
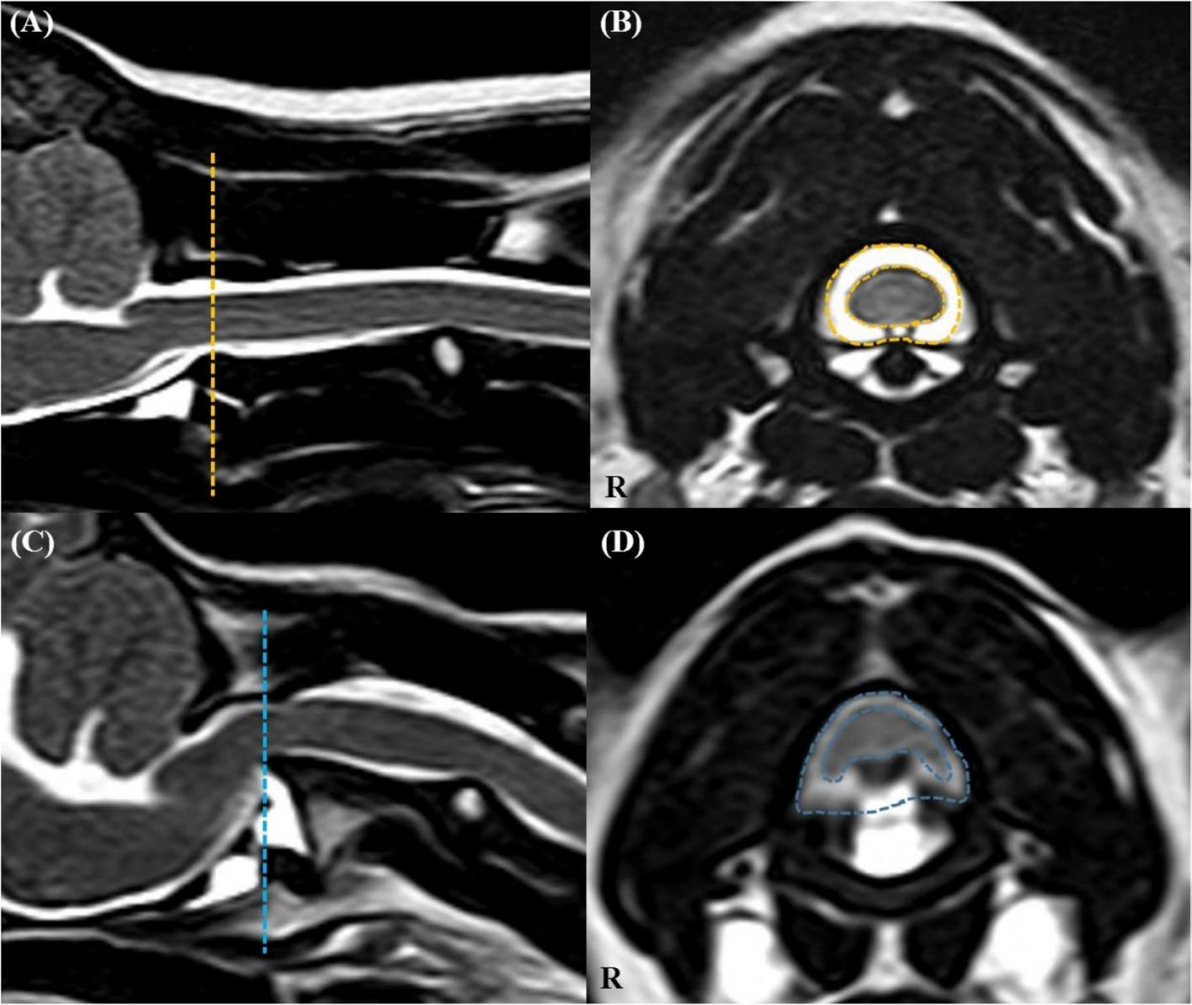
Comparison of the CSA ratio (**A**) and the fat ratio (**B**) of the SOMs between groups. Error bars illustrate that the CSA ratio of SOMs of normal dogs is higher than that of AAI dogs (**A**) and that group A has the highest the fat ratio of SOMs, followed by group B, with the lowest in normal dogs (**B**)

The measurements of SOMs are summarized in Table 2. Total CSA of the SOMs (*P* = 0.027) and CSA of the atlas (*P* = 0.01) increased as the weight of normal dogs increased. However, there was no correlation between body weight and the CSA ratio of SOMs in normal dogs (*P* = 0.268). The CSA ratio of SOMs (ratio of the CSA of the SOMs to the atlas) between normal and AAI dogs was significantly different (*P* = 0.029), but there was no statistical significance between the AAI groups (*P* = 0.836). There were statistical differences in the fat ratio of SOMs (ratio of the intensity of the SOMs to fat) between normal and AAI dogs (*P* = 0.044), and between AAI groups (*P* = 0.035) (Fig. 1). There was no significant correlation between the duration of neurological symptoms and the CSA ratio of SOMs (*P* = 0.951), or between the duration of neurological symptoms and the fat ratio of SOMs (*P* = 0.245), in AAI dogs. The ratio of spinal cord (ratio of the spinal cord to the canal) between normal and AAI dogs was significantly different (*P* = 0.001), but there was no significant difference between the AAI groups (*P* = 0.836). There was no statistical significance in the correlation between the ratio of spinal cord and the CSA ratio of SOMs, or between the ratio of spinal cord and the fat ratio of SOMs in AAI dogs. There was no correlation between all muscle parameters and age, and there were no significant differences between sexes in all groups.

**Table 2:** Ratio of spinal cord, CSA ratio and fat ratio of SOMs in normal and AAI dogs

Group		Ratio of spinal cord		CSA ratio of SOMs		Fat ratio of SOMs	
Normal		0.44 ± 0.04^a^		1.55 ± 0.23^b^		0.30 ± 0.05^c^	
AAI	A	0.3 ± 0.1	0.31 ± 0.12	1.29 ± 0.33	1.28 ± 0.38	0.35 ± 0.06^d^	0.37 ± 0.05
	B		0.32 ± 0.93		1.29 ± 0.29		0.33 ± 0.06

Data are expressed as the mean ± standard deviation. ^a^Significant difference in ratio of spinal cord to canal between normal and AAI dogs (*P* = 0.001). ^b^Significant difference in the ratio of CSA of SOMs to atlas between normal and AAI dogs (*P* = 0.029). ^c^Significant difference in the ratio of the intensity of SOMs to fat between normal and AAI dogs (*P* = 0.044). ^d^Significant difference in the ratio of the intensity of SOMs to fat between group A and B of AAI dogs (*P* = 0.035)

Intra-class correlation coefficient (ICC) regarding measurements was excellent (CSA of the spinal canal: 0.90–0.99; CSA of the spinal cord: 0.96–0.99; CSA of SOMs: 0.93–0.99; intensity of SOMs: 0.99).

## Discussion

The SOMs are three groups of short muscles that cover all surfaces of the atlas and axis. In dogs, the SOMs consists of rectus capitis dorsalis, oblique capitis caudalis, and oblique capitis cranialis [15]. Contraction of the rectus capitis dorsalis and the obliquus capitis cranialis muscles results in an extension of the head. The obliquus capitis caudalis muscle, which attaches laterally to the atlas wings and extends cranially to the spinous process of the axis, is one of the main muscles allowing rotation of the AAJ [16]. As they contribute to the movement of the AAJ, they could affect the stability in this region, and be affected by diseases affecting it.

In this study, the SOMs of AAI dogs appeared smaller and showed increased fat infiltration as compared to those of normal dogs, similar to the results from human studies on the relationship between pain and muscles [10]. This is also consistent with the findings on the epaxial muscles of the TL region in dogs [13]. The smaller SOMs in AAI dogs could be a sign of denervation atrophy; the painful and compressive characteristics and increased fat infiltration into the muscles is likely due to disuse and denervation [13, 17]. However, there is a disagreement between human studies on the influence of pain on muscles; for example, Yuan et al. (2017) reported a relationship between hypertrophy of this muscle and chronic headaches, while Hvedstrup et al. (2020) reported no association between pain and muscle properties [12, 18]. Skeletal muscles and bone share common embryological origins from mesodermal cell populations [19], and it has been reported that congenital abnormalities of bones in this region, such as incomplete ossification of the atlas, are associated with AAI [20]; therefore, it is possible that AAI dogs have congenital abnormalities of the SOMs as well as the bones.

Previous studies in dogs have reported that the severity of clinical symptoms had a significant effect on fat infiltration [13]. However, in this study, among AAI dogs, fat infiltration was more severe in group A dogs with mild symptoms than in group B dogs with severe symptoms. It has been reported that  fat infiltration in epaxial muscles increases with age in dogs [21]. In this study, group A was significantly older (7.58 years ± 3.08) than group B (2.56 years ± 1.49, *P* = 0.001). Therefore, it is possible that the fat infiltration identified in group A is age related, rather than associated with AAI. Although this study only evaluated a small number of dogs, there was no correlation between the fat ratio of muscles and age. In the long term, ongoing effects of pain and inflammatory mechanisms exert additional effects on muscle structure, such as atrophy, fatty infiltration, fibrosis, and function [22]. It is possible that these chronically altered SOMs reduce movement in AAJ, so that group A dogs showed mild symptoms for a long time, despite the AAI. To confirm this possibility, future studies are required to compare the histology of SOMs between normal and AAI dogs. However, intramuscular fat is reliably quantified using the signal intensity obtained by MRI [13, 14, 23]. The T1 relaxation time for fat tissue is short, and the signal intensity is high compared with skeletal muscle on the T1-weighted images; therefore, fat appears hyperintense relative to muscle, which is hypointense [23]. T1-weighted images have been used successfully to evaluate fat infiltration into various muscles in humans and dogs [13, 24–26]. Moreover, it has reported that increased fat infiltration and decreased muscle volume on T1-weighted images correlated well with subsequent histological analysis in isolated skeletal muscles of dogs [27]. Therefore, although histological confirmation was not possible, the method of quantifying fat infiltration used in this study may be useful as an index to non-invasively evaluate changes of the SOMs in AAI dogs.

There was no difference in the CSA of the muscle between the AAI groups in this study. It has been reported that nerve lesions affecting the multifidus CSA atrophy progress within three days [28]; most AAI dogs in this study showed neurological symptoms for more than three days; therefore, the possibility that there is no significant difference in the CSA of muscles in the AAI groups was considered.

This study identified a difference in the ratio of spinal cord between normal and AAI dogs. The spinal cord compression in AAI dogs may have affected atrophy and fat infiltration in the SOMs, similar to the results on the epaxial muscles in dogs [13]. However, the correlations between spinal cord compression and muscle atrophy, and between spinal cord compression and fat infiltration in AAI dogs were not statistically significant. A study on humans suggested that the degree of instability in AAI can be underestimated by MRI when compared with radiology [29]. All MRI in this study were taken in a neutral, not a flexed position; therefore, it is highly likely that spinal cord compression due to the degree of instability was not reflected in the measurement.

The main limitation of this study was that the number of dogs in each group was small, and therefore, we used a non-parametric test. Future studies are required with larger groups to investigate the SOMs in dogs. Due to ethical reasons, we were unable to conduct a subsequent histological verification for the muscle changes identified by MRI. In this study, intra-class reproducibility was verified; however, there was no inter-class reproducibility verification because an assessor requires training on MRI images and SOM anatomy for a significant time period. In this regard, further research related to inter-class verification according to the training level is needed for measurement of the muscles. Since the increase in the CSA of the SOMs according to body weight was identified even within toy-breed dogs with ≤ 5 kg, to compensate for possible discrepancies in body weight, the size of the SOMs was evaluated using the CSA ratio of muscles, which was not correlated with body weight because the disc measurement method used in other studies cannot be used in this area [13]. Despite the above limitations, this study provides fundamental quantitative information about MRI of the SOMs in normal and AAI dogs.

## Conclusions

This study found that AAI dogs had significantly smaller CSA of the SOMs and greater fat infiltration in the muscles compared to normal dogs. AAI dogs with mild neurological symptoms for a long period had greater fat infiltration in the muscles compared to AAI dogs with severe neurological symptoms. The muscle changes are most likely due to spinal cord compression from instability, however, since the possibility that chronic changes of the muscle may play an additional role in maintaining stability in this region cannot be excluded, larger group studies and histological studies of the muscles are needed. This study provides fundamental quantitative information of the SOMs in normal and AAI dogs.

## Methods

Two groups of client-owned small dogs (≤ 5 kg) admitted to Time Animal Hospital (Daejeon, South Korea) from March 2020 to June 2021 were prospectively studied. The first group consisted of 13 normal dogs that were defined as healthy based on physical, complete blood cell (CBC) counts, serum biochemistry, and cerebrospinal fluid (CSF) analyses. None of these dogs showed cervical neurological symptoms. The dogs were normal, except for some with vision loss or presumptively idiopathic seizures. The second group of dogs included 13 dogs with AAI. Physical and neurological examinations, CBC counts, and serum biochemical analyses were also performed in all AAI dogs. To confirm the abnormality of the dens, computed tomography examination was also performed for all AAI dogs. The time of onset of the clinical signs and severity was recorded. By use of a grading system [30], the neurological status of the AAI dogs was graded from 1 to 5 as follows: grade 1: no neurological impairment; grade 2: ambulatory with paresis; grade 3: non-ambulatory paresis; grade 4: quadriplegia and grade 5: dead. A comparison between AAI dogs was made by dividing them into two groups: group A (mild symptoms, grades 1–2) and group B (severe symptoms, grades 3–5).

Cervical MRI examinations were performed with a 1.5-Tesla (Intera, Philips Healthcare, Netherlands) system under general anaesthesia. Anaesthesia was induced in each dog with intravenous propofol (6 mg/kg, Provive 1%, Myungmoon Pharm. Co., Korea) and maintained with isoflurane (Ifran, Hana Pharm., Korea) and oxygen. Dogs were positioned in ventral recumbency, with the forelimbs pulled caudally and a straight spinal alignment, without any pressure on the cervical muscles on the eight-channel knee coil. Transverse MRI settings for measurements were as follows: slice thickness of 2.0 mm, gap of 0.2 mm, T1 weighted (TR = 2000 ms; TE = 10 ms) and T2 weighted (TR = 3000–4000 ms; TE = 100 ms). Dorsal and sagittal T2-weighted and sagittal T1-weighted images were also obtained of the surrounding structures, for reference.

**Fig. 2:**
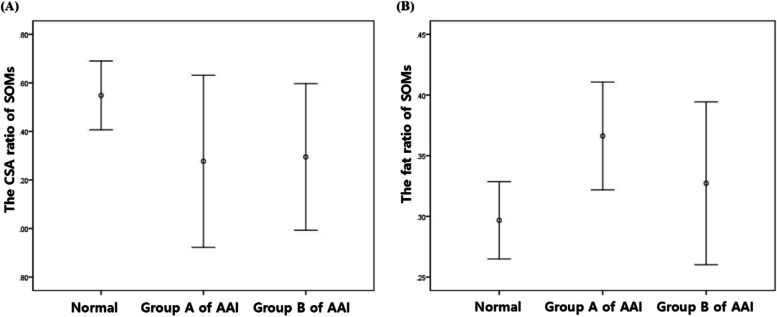
The measurements of the CSA and the intensity of SOMs and the intensity of fat on the left side at C1-2 in a normal dog. Manually traced ROI: the CSA of the SOMs (orange line) and the CSA of atlas (red line). The ratio of the intensity of muscle to fat was calculated by dividing the intensity of the muscle (black arrow) by the intensity of the fat (white arrow): 556/1768 = 0.31

**Fig. 3:**
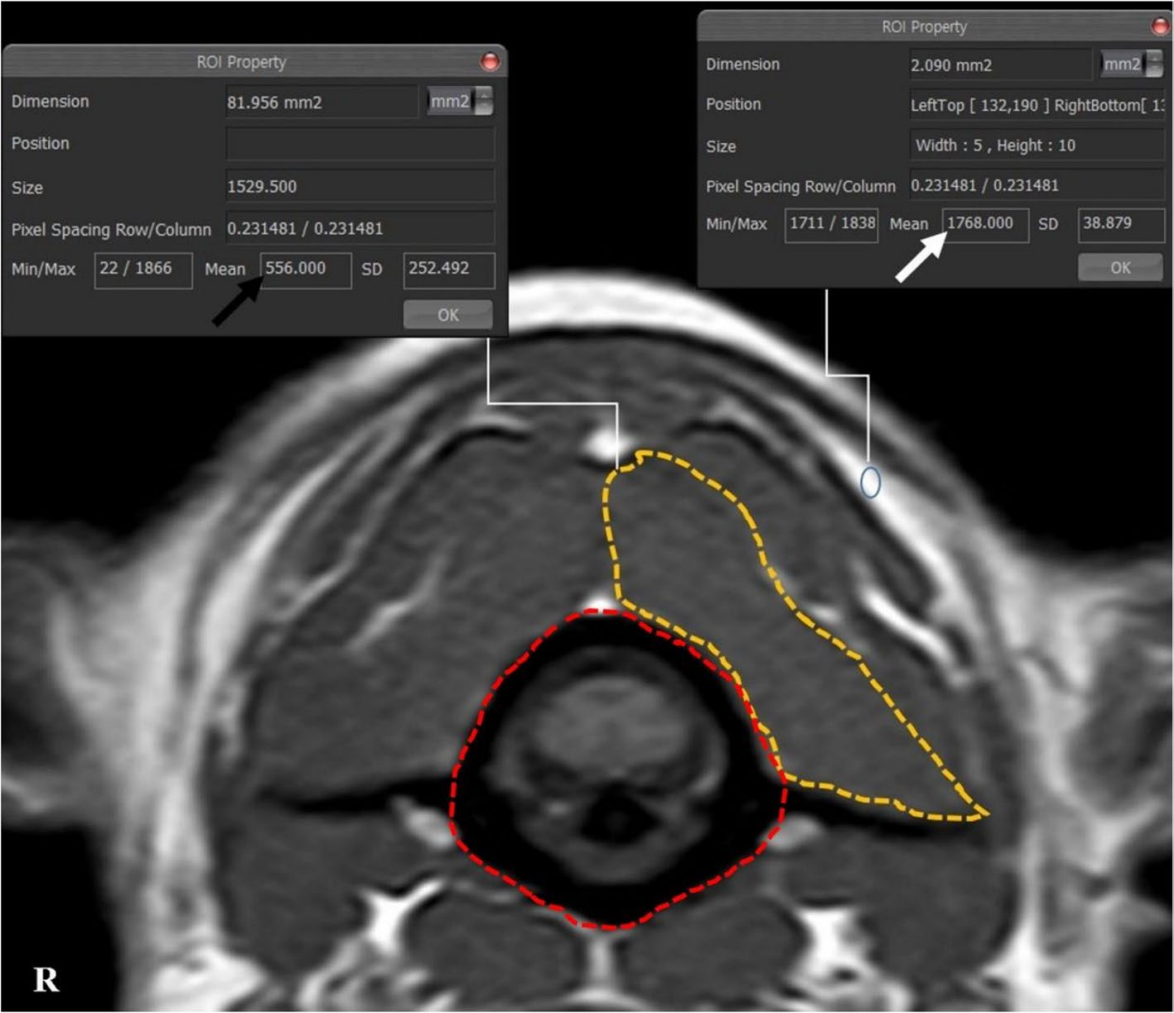
The CSA of the spinal canal (CSF column) and cord in a normal dog (**A**, **B**) and an AAI dog (**C**, **D**). On the transverse T2-weighted images (**B**, **D**), CSA of the spinal canal (outer line) and CSA of the spinal cord (inner line) were measured at C1-2 level of the corresponding of line at the sagittal T2-weighted images (**A**, **C**)

Images were analysed via picture archiving and the use of a communication system (PACSPLUS, Medical Standard, Korea). Measurements followed the methods described in previous reports on dogs and humans [13, 23, 31]. On the transverse T-weighted images, the CSAs of the SOMs (sum of rectus capitis dorsalis, oblique capitis caudalis, and oblique capitis cranialis) were measured bilaterally at C1-2 by manually drawing a region of interest (ROI), tracing the outer margin of the muscles (Fig. 2). The muscles were traced based on the visible bony landmarks, canine anatomy literature [32–35] and a previous anatomic MRI study of this region [36]. Possible intermuscular fat was excluded. When the boundary between the SOMs and other muscles (semispinalis and longissimus muscle) was unclear, the ROI was defined through the middle of these regions to allow a reasonable approximation of the muscle’s anticipated boundary [37]. Total CSA of the SOMs was calculated by summing the bilateral measurements. To compensate for possible discrepancies in body weight, the CSA of the atlas (C1) was traced based on the outer margin of the atlas body on the same images, and the ratio of the CSA of the SOMs to the atlas (CSA ratio of SOMs) was calculated by dividing the total CSA of the bilateral muscles by the CSA of the atlas. To evaluate presumptive fat infiltration in the muscles, the signal intensity of the muscles was measured from the same ROIs, and the signal intensity of fat (approximately 2 mm^2^) was also measured at the whitest spot (hyperintense) on the same T1-weighted images (Fig. 2). Ratio of the intensity of the SOMs (mean intensity of bilateral muscles) to fat (fat ratio of SOMs) was calculated by dividing the intensity of the muscles by the intensity of the fat. To evaluate the relationship between the degree of compression of the spinal cord and these values, the ratio of the spinal cord to the canal (ratio of spinal cord) was determined by dividing the CSA of the spinal cord by the CSA of the spinal canal (CSF column) on the transverse T2-weighted images, with reference to the T1-weighted images (Fig. 3).

The values were measured three times at an interval of one week, and the assessor (NS Lee) was blinded to the earlier values.

Statistical analyses of the data were performed using SPSS software (IBM SPSS Statistics 22, IBM Corporation, USA). Kendall’s Tau was used to identify the significant correlations between the values and body weight, age, the compression rate, and the duration of neurological symptoms. The Mann-Whitney *U*-test was used to compare between the values of normal and AAI dogs, and between the values of the groups within AAI and sex. Reproducibility was assessed using the ICC: a value close to 1 indicates excellent agreement. A *P*-value of < 0.05 was considered statistically significant.

## Data Availability

The datasets used and/or analysed during the current study are available from the corresponding author on reasonable request.

## Abbreviations

AAI: Atlantoaxial instability
AAJ: Atlantoaxial joint
SOMs: Suboccipital muscles
MRI: Magnetic resonance imaging
CSA: Cross-sectional area
ICC: Intra-class correlation coefficient
TL: Thoracolumbar
CBC: Complete blood cell
CSF: Cerebrospinal fluid
ROI: Region of interest
