# Correction to: Comparison of cross-sectional area and fat infiltration of suboccipital muscles between normal dogs and dogs with atlantoaxial instability

**DOI:** 10.1186/s12917-022-03177-9

**Published:** 2022-03-01

**Authors:** Namsoon Lee, Munsu Yun, Junghee Yoon

**Affiliations:** 1 Time Animal Medical Center, 57, Dunsan-ro, Seo-gu, Daejeon, 35233 South Korea; 2 College of Veterinary Medicine, Research Institute for Veterinary Science, Seoul National University, Gwanak-ro, Gwanak-gu, Seoul, 08826 South Korea

**Correction to: BMC Vet Res 18, 46 (2022)**

**https://doi.org/10.1186/s12917-021-03132-0**

Following the publication of the original paper [1], authors spotted the error pertaining to mismatched figure images and captions/legends. Please find the following corrected figures and captions/legends.

Figures 1, 2, 3

**Fig. 1:**
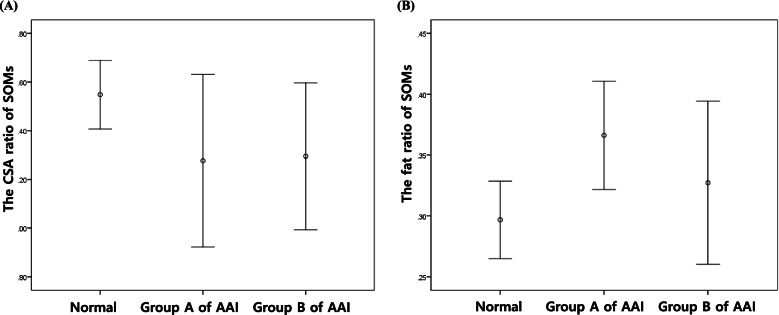
Comparison of the CSA ratio (**A**) and the fat ratio (**B**) of the SOMs between groups. Error bars illustrate that the CSA ratio of SOMs of normal dogs is higher than that of AAI dogs (**A**) and that group A has the highest the fat ratio of SOMs, followed by group B, with the lowest in normal dogs (**B**)

**Fig. 2:**
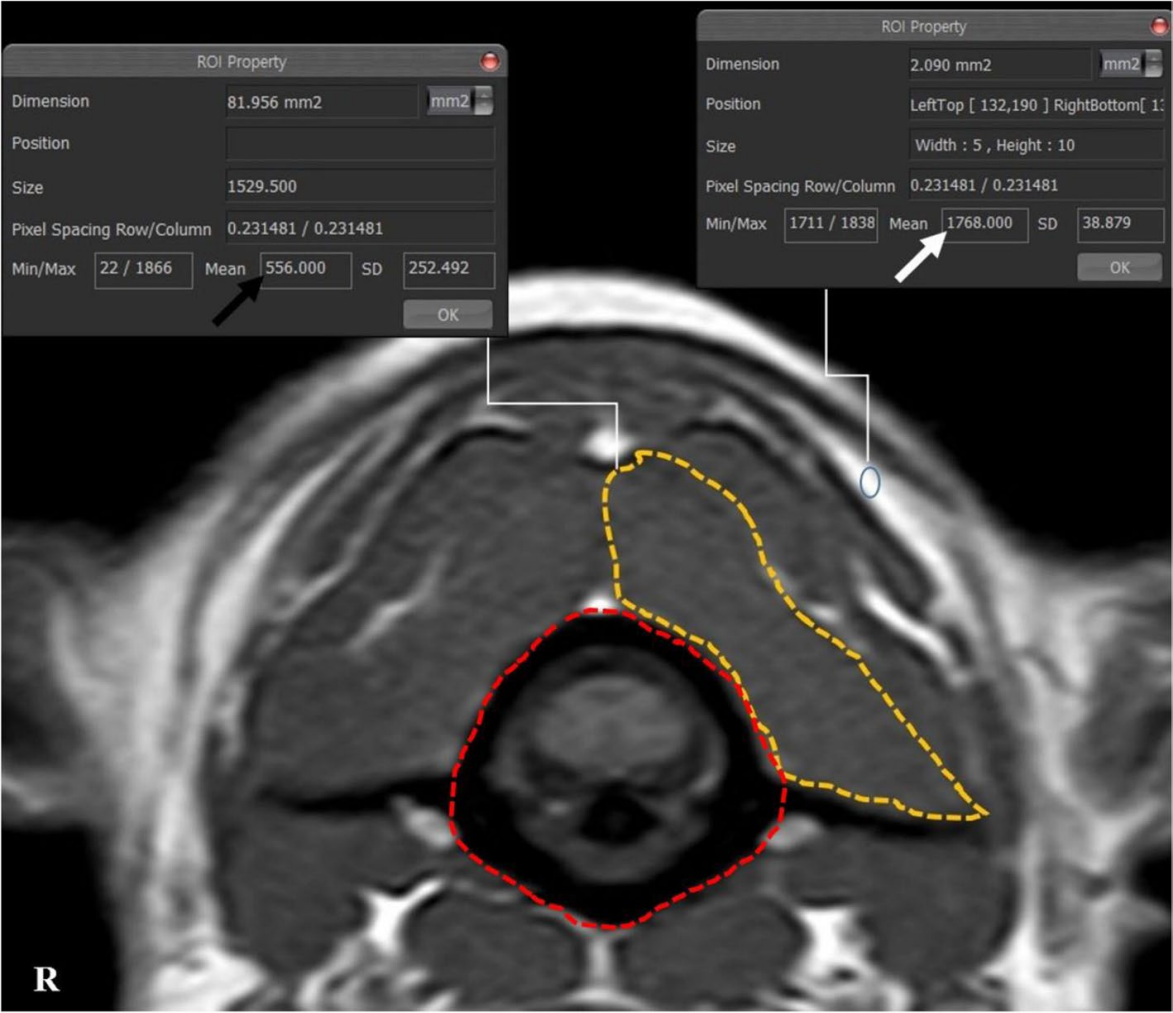
The measurements of the CSA and the intensity of SOMs and the intensity of fat on the left side at C1-2 in a normal dog. Manually traced ROI: the CSA of the SOMs (orange line) and the CSA of atlas (red line). The ratio of the intensity of muscle to fat was calculated by dividing the intensity of the muscle (black arrow) by the intensity of the fat (white arrow): 556/1768=0.31

**Fig. 3:**
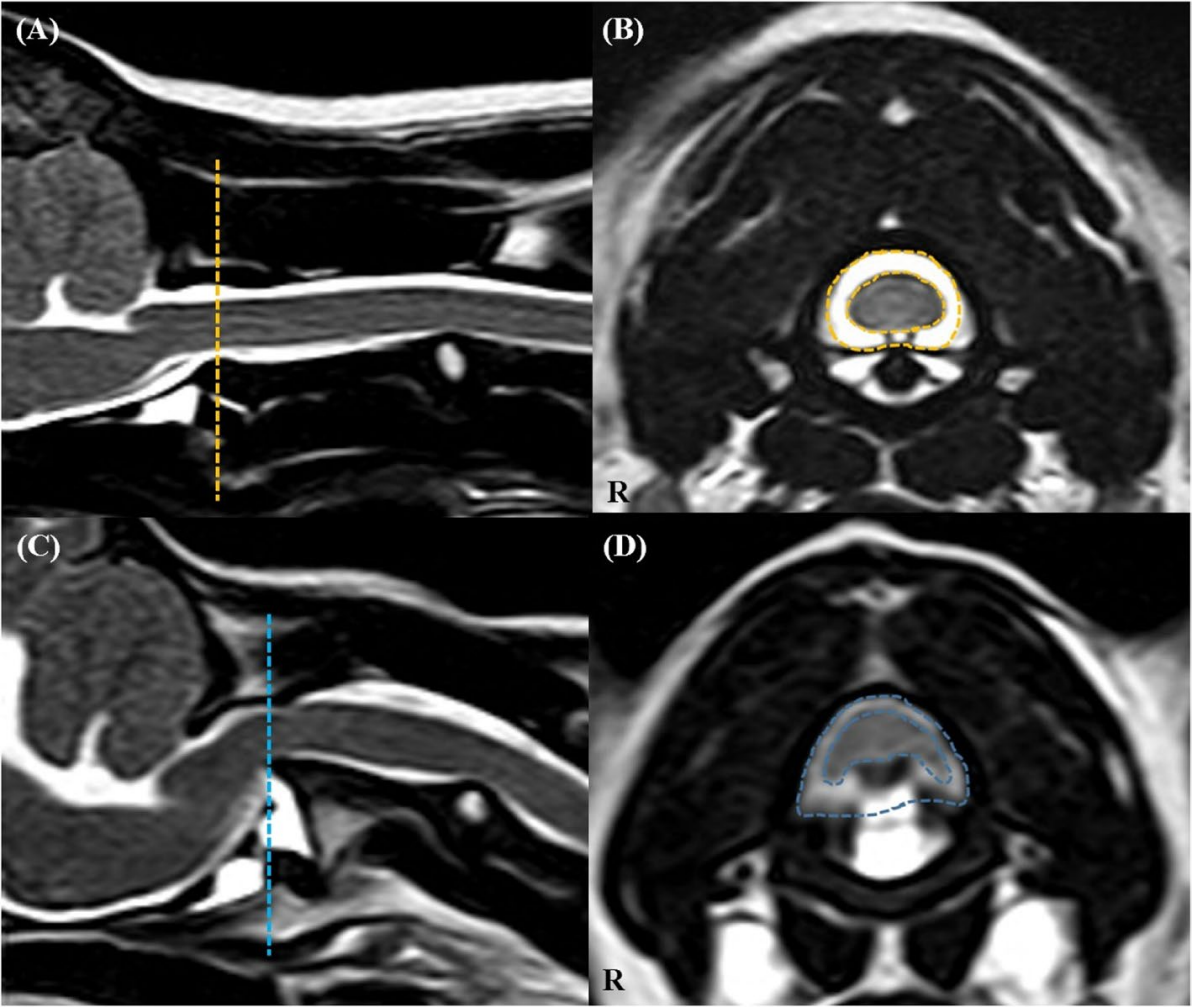
The CSA of the spinal canal (CSF column) and cord in a normal dog (**A**, **B**) and an AAI dog (**C**, **D**). On the transverse T2-weighted images (**B**, **D**), CSA of the spinal canal (outer line) and CSA of the spinal cord (inner line) were measured at C1-2 level of the corresponding of line at the sagittal T2-weighted images (**A**, **C**)

The original article has been corrected.
